# Differential Impact of Body Mass Index in Hip Arthroscopy: Obesity Does Not Impact Outcomes

**DOI:** 10.31486/toj.22.0077

**Published:** 2023

**Authors:** Misty Suri, Arjun Verma, Mohammed Asad Khalid, Michael Nammour, Deryk Jones, Brian Godshaw

**Affiliations:** ^1^ Ochsner Sports Medicine Institute, Jefferson, LA; ^2^ The University of Queensland Medical School, Ochsner Clinical School, New Orleans, LA

**Keywords:** *Arthroscopy*, *body mass index*, *hip joint*

## Abstract

**Background:** Hip arthroscopy is commonly used for the treatment of hip pathologies. As population obesity rates continue to increase, elucidating the impact of body mass index (BMI) on hip arthroscopy outcomes is essential. This investigation was conducted to quantify the effects of BMI on hip arthroscopy outcomes.

**Methods:** We conducted a retrospective medical records review of 459 patients undergoing hip arthroscopy at a single center from 2008 to 2016. The Harris Hip Score (HHS) and 2 component scores of the 12-Item Short Form Survey—the physical component score (PCS-12) and the mental component score (MCS-12)—were used to measure outcomes. Patients were stratified into 4 cohorts based on their BMI: underweight (BMI <18.5 kg/m^2^), normal weight (BMI 18.5 to 24.9 kg/m^2^), overweight (BMI 25.0 to 29.9 kg/m^2^), and obese (BMI ≥30.0 kg/m^2^).

**Results:** At 1 and 2 years postoperatively, all cohorts experienced statistically significant improvements in the HHS and PCS-12. At 3 years postoperatively, statistically significant improvements were seen in the HHS for all cohorts; in the PCS-12 for the normal weight, overweight, and obese cohorts; and in the MCS-12 for the normal weight cohort. Intercohort differences were not statistically significant at 1, 2, or 3 years postoperatively.

**Conclusion:** In our population, BMI did not have statistically significant effects on patient outcome scores following hip arthroscopy. All patient cohorts showed postoperative improvements, and differences between BMI cohorts were not statistically significant at any postoperative time point.

## INTRODUCTION

In recent decades, global obesity rates have continued to increase. Since 1975, obesity rates have more than tripled, and as of 2016, an estimated 39% of adults were overweight and 13% were obese.^1^ If current trends continue, an estimated 65 million additional Americans will be obese by 2030, raising the obesity rate to 42%.^2,3^ The financial impact of obesity cannot be understated, as, by 2030, the estimated loss of productivity related to obesity could reach $580 billion annually in the United States.^3^ Annual medical costs in obese individuals are, on average, 42% higher than those for healthy-weight individuals.^2^ Interestingly, although the financial and health-related ramifications of obesity have been widely examined, the debate continues regarding the optimal measures of obesity.^4^

Body mass index (BMI) is calculated using a patient's height and weight and is used to classify patients as underweight (BMI <18.5 kg/m^2^), normal weight (BMI 18.5 to 24.9 kg/m^2^), overweight (BMI 25.0 to 29.9 kg/m^2^), and obese (BMI ≥30.0 kg/m^2^).^5^ BMI is often used as an indicator of a patient's current health and the possibility of future health problems. Importantly, while BMI is not a perfect measurement of obesity, it has been shown to possess high specificity and to be clinically equivalent to other proposed methods for identifying obesity.^6^ Obesity has been shown in the orthopedic literature to be associated with worse postoperative outcomes and to increase the risk of complications. Harrison et al showed that after partial meniscectomy or arthroscopic knee debridement, obese patients had worse physical functioning as measured by the 36-Item Short Form Survey compared to nonobese patients.^7^ Warrender et al found that after arthroscopic rotator cuff repair, obese patients had worse functional outcomes, longer operative times, and longer hospital stay compared to nonobese patients.^8^ A meta-analysis by Yuan et al showed a 2-fold increased risk of surgical site infection in obese patients.^9^

Hip arthroscopy is commonly used to treat hip pathologies such as labral tears, femoroacetabular impingement, and loose bodies. The goals of hip arthroscopy are to alleviate symptoms, improve hip function, and delay the progression of hip osteoarthritis. The use of hip arthroscopy has continued to increase in recent years, with an 18-fold increase in hip arthroscopy cases from 1999 to 2009.^10^ Clohisy et al found that nearly 42% of patients undergoing hip arthroscopy for femoroacetabular impingement are overweight or obese.^11^ As hip arthroscopy has grown in popularity and the obesity rate has continued to rise, evaluation of hip arthroscopy outcomes in the obese patient is a growing need.

Although a significant amount of orthopedic literature is available on obesity and outcomes after knee and shoulder arthroscopy and arthroplasty, few studies of hip arthroscopy and obesity have been done. These studies have suggested poorer outcomes in patients receiving hip arthroscopy as BMI increases.^12-16^ Given the increasing popularity of this procedure and rising obesity rates, we conducted this investigation to examine and quantify the differential effect of BMI and obesity on the outcomes of patients undergoing hip arthroscopy.

## METHODS

After approval from the institutional review board, we conducted a retrospective review of prospectively collected data for all patients undergoing hip arthroscopy from 2008 to 2016 at a single institution. All procedures were performed by a single, fellowship-trained surgeon (MS). Inclusion criteria included hip arthroscopy with a minimum of 1 year of clinical follow-up. Exclusion criteria included revision hip arthroscopy and clinical follow-up of <1 year.

Demographic data obtained from the medical records were sex, age at time of hip arthroscopy, and BMI. We stratified patients into 4 cohorts based on their BMI at the time of surgery: underweight (BMI <18.5 kg/m^2^), normal weight (BMI 18.5 to 24.9 kg/m^2^), overweight (BMI 25.0 to 29.9 kg/m^2^), and obese (BMI ≥30.0 kg/m^2^).

We assessed outcomes using 2 validated methods. We used the Harris Hip Score (HHS), a commonly used questionnaire for assessing hip dysfunction, to assess patient pain, function, activity, and various physical examination data. The HHS has a maximum score of 100, with higher values indicating better outcomes.^17^ We also used 2 component scores of the 12-Item Short Form Survey (SF-12)—the physical component score (PCS-12) and the mental component score (MCS-12)—to assess patients’ physical and mental health. The SF-12 and its components also have a maximum score of 100, with higher scores indicating better physical and mental health.^18^ Patients completed the questionnaires at their clinical appointments preoperatively and at 6 weeks, 3 months, 6 months, 1 year, 2 years, and 3 years postoperatively.

SAS version 9.4 for Windows (SAS Institute, Inc.) was used for all statistical analyses. Tests were performed with a significance level of α=0.05, and any values were considered statistically significant if *P*<α. Analysis of variance, Wilks lambda, and solution for fixed effects were used to assess outcome measures based on BMI and between BMI cohorts. Intracohort *P* values were calculated using paired *t* tests to compare preoperative and postoperative outcome scores.

## RESULTS

Of the 484 patients who underwent hip arthroscopy during the study period, 25 met the exclusion criteria. Of the remaining 459 patients with recorded preoperative BMIs, 46.4% were in the normal weight cohort, and the average BMI for all patients was 25.7 kg/m^2^. The majority of patients were female (59.3%), and the average age of all patients was 35.6 years (Table 1).

**Table 1.** Demographic Information, n=459

Variable	Value
Age, years, mean	35.6
Sex	
Female	272 (59.3)
Male	187 (40.7)
Body mass index, kg/m^2^, mean	25.7
Body mass index cohorts	
Underweight	16 (3.5)
Normal weight	213 (46.4)
Overweight	147 (32.0)
Obese	83 (18.1)

Notes: Data are presented as n (%) unless otherwise indicated. Body mass index cohorts were defined as follows: underweight, <18.5 kg/m^2^; normal weight, 18.5 to 24.9 kg/m^2^; overweight, 25.0 to 29.9 kg/m^2^; obese, ≥30.0 kg/m^2^.

**Figure 1.**
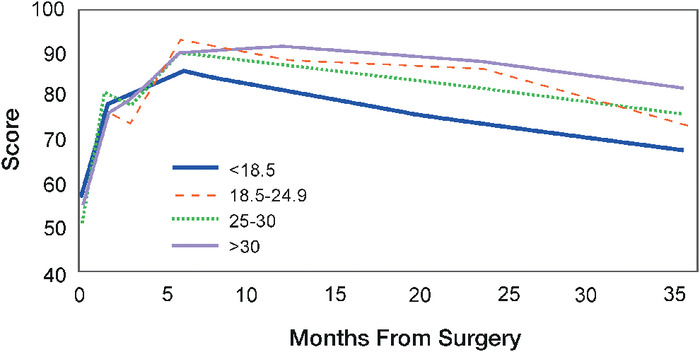
Mean scores for the Harris Hip Score (HHS) from baseline (preoperatively) to 36 months postoperatively stratified by body mass index cohort. The HHS, which is used to measure hip functionality and pain, has a maximum score of 100, with higher scores indicating better outcomes.

The HHS results for each BMI cohort are shown in Table 2 and Figure 1. The underweight group had the highest preoperative HHS of 58.0, and the overweight group had the lowest initial score at 50.6. In the underweight, normal weight, and overweight groups, the highest HHS value was at 6 months postoperatively and then steadily declined in the subsequent years. The obese group had its highest HHS value at 1 year postoperatively, followed by a steady decline. At the 3-year postoperative time point, the obese group had the highest overall HHS, and the underweight group had the lowest overall improvement. Compared to their preoperative scores, all BMI cohorts had statistically significant improvements in the HHS at 1, 2, and 3 years postoperatively. However, the intercohort differences in HHS between BMI cohorts were not statistically significant at any of the time points.

**Table 2.** Average Preoperative and Postoperative Scores Stratified by Body Mass Index Cohort

		Postoperative Follow-Up Time Point					
Outcome Measure/Body Mass Index Cohort	Preoperative Baseline	6 weeks	3 months	6 months	1 year	2 years	3 years
**Harris Hip Score**							
Underweight	58.0	78.1 (0.020)	80.2 (0.001)	85.9 (0.076)	81.0 (0.011)	73.2 (0.008)	68.2 (0.046)
Normal weight	56.3	76.7 (<0.001)	74.0 (<0.001)	92.5 (<0.001)	88.5 (<0.001)	86.3 (<0.001)	73.9 (<0.001)
Overweight	50.6	80.5 (<0.001)	77.9 (<0.001)	89.8 (<0.001)	87.4 (<0.001)	82.0 (<0.001)	75.9 (<0.001)
Obese	54.7	75.9 (<0.001)	79.5 (<0.001)	90.2 (<0.001)	91.7 (<0.001)	87.8 (<0.001)	82.2 (<0.001)
Intercohort *P* value^a^	0.245	0.914	0.989	0.576	0.884	0.444	0.448
**Physical Component Score of 12-Item Short Form Survey**							
Underweight	37.1	38.0 (0.494)	44.2 (0.008)	43.7 (0.016)	46.9 (0.024)	48.4 (0.022)	49.0 (0.061)
Normal weight	35.6	38.2 (0.002)	44.9 (<0.001)	49.4 (0.001)	49.6 (<0.001)	50.1 (<0.001)	51.4 (<0.001)
Overweight	34.6	38.4 (0.001)	43.4 (<0.001)	46.7 (<0.001)	48.3 (<0.001)	49.1 (<0.001)	51.8 (<0.001)
Obese	34.2	47.5 (0.079)	42.9 (<0.001)	46.7 (<0.001)	49.2 (<0.001)	51.1 (<0.001)	53.6 (<0.001)
Intercohort *P* value^a^	0.542	0.186	0.639	0.796	0.758	0.765	0.661
**Mental Component Score of 12-Item Short Form Survey**							
Underweight	48.0	52.4 (0.049)	53.4 (0.059)	47.7 (0.353)	58.0 (0.026)	57.2 (0.022)	55.5 (0.056)
Normal weight	49.5	53.4 (0.001)	55.1 (<0.001)	54.6 (<0.001)	54.9 (<0.001)	54.5 (<0.001)	55.4 (<0.001)
Overweight	51.2	53.3 (0.004)	55.0 (<0.001)	54.3 (0.010)	54.9 (0.049)	55.4 (0.071)	56.3 (0.119)
Obese	50.9	53.7 (0.017)	54.9 (0.030)	54.9 (0.027)	55.9 (0.010)	56.4 (0.079)	59.0 (0.095)
Intercohort *P* value^a^	0.519	0.984	0.927	0.269	0.669	0.671	0.318

^a^Intercohort *P* values calculated by analysis of variance to compare the mean values of the 4 body mass index cohorts.

Notes: Data are presented as mean score (*P* value compared to preoperative score) unless otherwise indicated. The Harris Hip Score, the physical component score of the 12-Item Short Form Survey, and the mental component score of the 12-Item Short Form Survey each has a maximum score of 100, with higher values indicating better outcomes. Body mass index cohorts were defined as follows: underweight, <18.5 kg/m^2^; normal weight, 18.5 to 24.9 kg/m^2^; overweight, 25.0 to 29.9 kg/m^2^; obese, ≥30.0 kg/m^2^.

**Figure 2.**
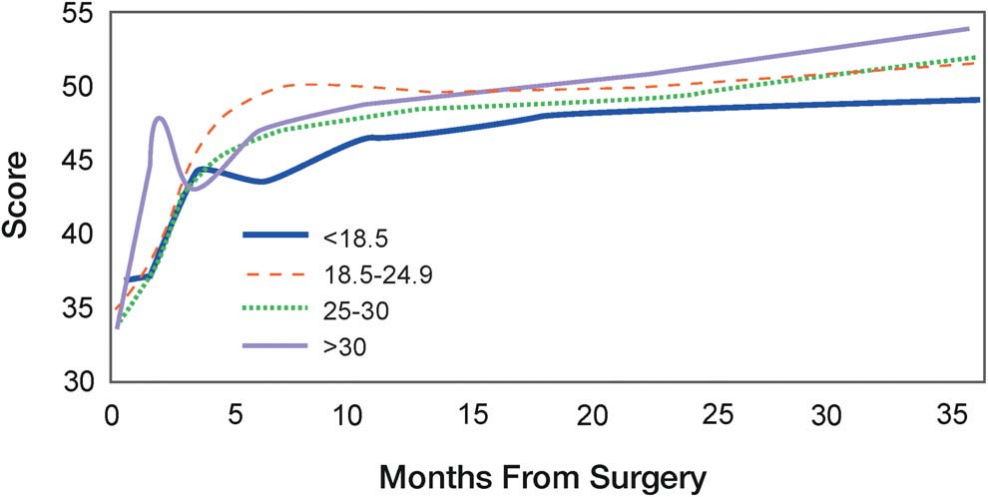
Mean scores for the physical component score (PCS-12) from baseline (preoperatively) to 36 months postoperatively stratified by body mass index cohort. The PCS-12, which is the physical health subcomponent of the 12-Item Short Form Survey, has a maximum score of 100, with higher scores indicating better outcomes.

PCS-12 results for each BMI cohort are shown in Table 2 and Figure 2. The underweight group again had the highest baseline PCS-12 at 37.1, while the obese group had the lowest score at 34.2. The PCS-12 increased at each time point until 3 years postoperatively for the normal weight and overweight groups. The underweight group exhibited similar score increases at most follow-up time points, except for a decline in the PCS-12 at 6 months postoperatively. Likewise, the PCS-12 for the obese cohort increased at most follow-up points but exhibited declines at 3 months and 6 months. At 3 years postoperatively, the obese group had the highest PCS-12 at 53.6, the largest improvement from the preoperative PCS-12, with an average 19.4-point improvement. In comparison, the overweight group had a 17.2-point improvement, the normal weight group had a 15.8-point improvement, and the underweight group had the least improvement at 11.9 points. Compared to preoperative scores, all BMI cohorts experienced statistically significant improvement in the PCS-12 at 3 months, 6 months, 1 year, and 2 years postoperatively. The normal weight, overweight, and obese groups also experienced statistically significant improvements in the PCS-12 at 3 years postoperatively compared to preoperative scores. At 3 years postoperatively, the mean score in the underweight group improved compared to preoperative values, but the difference was not statistically significant. The intercohort differences between BMI cohorts for the PCS-12 were not statistically significant at any time point.

**Figure 3.**
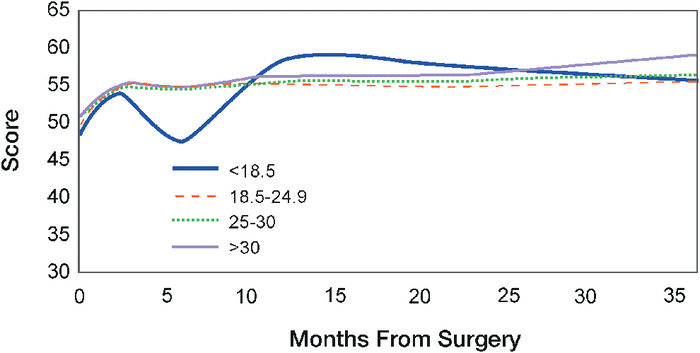
Mean scores for the mental component score (MCS-12) from baseline (preoperatively) to 36 months postoperatively stratified by body mass index cohort. The MCS-12, which is the mental health subcomponent of the 12-Item Short Form Survey, has a maximum score of 100, with higher scores indicating better outcomes.

MCS-12 results for each BMI cohort are shown in Table 2 and Figure 3. The overweight group had the highest initial score of 51.2, and the underweight group had the lowest initial score of 48.0. At 1 year postoperatively, all BMI cohorts experienced statistically significant improvements in the MCS-12. At 2 years postoperatively, the improvement of the underweight and normal weight groups remained statistically significant. By 3 years postoperatively, only the normal weight group achieved improvement of statistical significance. At 3 years postoperatively, the obese group had the highest MCS-12 at 59.0, the largest improvement from the preoperative MCS-12 with an average 8.1-point improvement. In comparison to baseline scores, the underweight group improved by 7.5 points, the normal weight group by 5.9 points, and the overweight group by 5.1 points. The intercohort differences in the MCS-12 were not statistically significant at any time point.

## DISCUSSION

This retrospective review provides the results of patient-reported outcomes from different BMI cohorts and demonstrates significant improvements in all outcome measures (HHS, PCS-12, and MCS-12) in each BMI cohort with no significant difference between groups at final follow-up.

Despite the paucity of orthopedic literature discussing the relationship between obesity and outcomes after hip arthroscopy, some studies have indicated that patients with higher BMIs have poorer outcomes following hip arthroscopy.

Gupta et al performed 2 studies evaluating the effect of obesity on hip arthroscopy outcomes.^12,14^ In a 2015 study, they compared patient-reported outcomes after hip arthroscopy from 62 obese patients and 124 controls.^12^ Their results showed that preoperatively, obese patients started with lower patient-reported outcome scores compared to nonobese patients, and at 2 years postarthroscopy, obese patients had significantly lower patient-reported outcome scores. However, statistically significant improvement was seen in both the obese and nonobese populations. The researchers concluded that both groups demonstrated significant improvement and that the change was similar between the 2 groups.^12^ In another 2015 study, Gupta et al conducted a cohort analysis of 680 patients to determine if obesity impacted postoperative outcome scores.^14^ They found that obese patients had lower preoperative and postoperative scores compared to nonobese patients, but both nonobese and obese patients showed substantial improvement.

Bech et al performed a systematic review of 3 studies on the outcomes of hip arthroscopy in obese patients compared with a nonobese cohort.^15^ They found that although obese patients obtained similar improvements postoperatively, obese patients had lower patient-reported outcome scores at follow-up, were 4.7 times more likely to require re-arthroscopy, and were 2.2 times more likely to require conversion to total hip replacement than nonobese patients. Bech et al concluded that because of the lower overall outcome scores and increased reoperation rate, hip arthroscopy should be used with caution in obese patients.^15^

Schairer et al conducted a retrospective population-based analysis of 7,351 patients to evaluate the conversion rate to total hip arthroplasty 2 years after hip arthroscopy.^19^ Their results showed that conversion to total hip arthroplasty was highest in patients 60 to 69 years old (35%) and that obese patients were more likely to undergo total hip arthroplasty within 2 years: 22.8% of obese patients vs 11.4% of non-obese patients (odds ratio 2.31, *P*<0.001). Of note, patients treated at low volume hip arthroscopy centers (<10 procedures performed annually) were significantly more likely to undergo total hip arthroplasty within 2 years than patients treated at medium volume (10 to 49 procedures performed annually) and high volume (>49 procedures performed annually) centers.^19^

Our study, however, showed that patients with higher BMIs enjoyed outcomes similar to those of patients with lower BMIs. Our results show that all BMI cohorts experienced improved patient-reported outcome scores at 1, 2, and 3 years postoperatively. Moreover, intercohort differences in patient-reported outcome scores showed no statistically significant differences at 1, 2, or 3 years postoperatively. In fact, this study found that the obese group had the greatest magnitude of improvement in all outcome measures. The obese group also had the highest HHS, PCS-12, and MCS-12 at 3 years postoperatively (Table 2). The underweight group had the least improvement in the HHS and PCS-12. These results suggest that obese patients do not experience worse outcomes at 1, 2, or 3 years postoperatively, a finding that differs from prior studies.

The senior lead author of this study (MS), based on the definitions in Schairer et al,^19^ is a high-volume surgeon who performs >50 hip arthroscopies annually. Thus, experience may have played a role in improved outcomes across all groups. Regardless, increased BMI did not appear to be a limiter of our patients’ long-term outcomes following hip arthroscopy as reported in other studies.

One of the strengths of this study is that all patients were treated in a single institution by the same surgeon under similar conditions, helping to decrease the risk of variability that may naturally occur by considering patients treated at a variety of institutions by multiple surgeons. Further, all patients were examined preoperatively and postoperatively by the treating physician using consistent assessment and physical examination, allowing for accurate documentation of patients’ progression throughout their course of treatment.

A weakness of this study is that it was conducted at a single center and is entirely retrospective. Moreover, we did not use specific indications for arthroscopy and did not delineate or assess patients’ expectations of postoperative activity levels. Another weakness is that we did not examine the rate of re-arthroscopy or conversion to total hip arthroplasty. Some of the previously cited articles discuss obesity as a risk factor for reoperation and for conversion to total hip arthroplasty.^12,14,15,20^ This study does not address these concerns, but based on the literature, treating hip surgeons should discuss with obese patients that they may be at increased risk for reoperation or conversion to total hip arthroplasty.

## CONCLUSION

The results of this study indicate no correlation between BMI and patient outcomes following hip arthroscopy in our patient population. These results are encouraging in that patients with higher BMIs may choose hip arthroscopy to treat a variety of hip pathologies while still expecting favorable outcomes regardless of body habitus.

## Notes

*This article meets the Accreditation Council for Graduate Medical Education and the American Board of Medical Specialties Maintenance of Certification competencies for Patient Care, Medical Knowledge, and Practice-Based Learning and Improvement.*
